# Gray matter correlates of impulsivity in psychopathy and in the general population differ by kind, not by degree: a comparison of systematic reviews

**DOI:** 10.1093/scan/nsab045

**Published:** 2021-04-09

**Authors:** Cole Korponay, Michael Koenigs

**Affiliations:** Basic Neuroscience Division, McLean Hospital, Belmont, MA 02478, USA; Department of Psychiatry, Harvard Medical School, Cambridge, MA 02215, USA; Department of Psychiatry, University of Wisconsin–Madison, Madison, WI 53719, USA

**Keywords:** impulsivity, gray matter, prefrontal cortex, striatum, psychopathy, healthy

## Abstract

A fundamental question in neuropsychiatry is whether a neurobiological continuum accompanies the behavioral continuum between subclinical and clinical traits. Impulsivity is a trait that varies in the general population and manifests severely in disorders like psychopathy. Is the neural profile of severe impulsivity in psychopathy an extreme but continuous manifestation of that associated with impulsivity in the general population (different by degree)? Or is it discontinuous and unique (different by kind)? Here, we compare systematic reviews of the relationship between impulsivity and gray matter in psychopathy and in the general population. The findings suggest that the neural profile associated with extreme impulsivity in psychopathy (increased gray matter in rostral and ventral striatum and prefrontal cortexes) is distinct from that associated with impulsivity in the general population (decreased gray matter in rostral and ventral prefrontal cortexes). Severe impulsivity in psychopathy may therefore arise from a pathophysiological mechanism that is unique to the disorder. These findings prompt the need for future studies to directly test the effect of group on the impulsivity–gray matter relationship in samples comprised of healthy individuals and individuals with psychopathy. The results caution against the use of community samples to examine impulsive psychopathic traits in relation to neurobiology.

## Introduction

Impulsive decisions are those that are made in haste, spurred by the prospect of an immediate reward or by impatience and/or made without sufficient regard for their long-term consequences. For individuals with a consistent propensity for impulsive decision-making—and especially for individuals with psychiatric conditions featuring clinical levels of impulsivity (Moeller *et al.*, 2001)—everyday functioning can become impaired to the point that quality of life suffers. In the general population, impulsivity has been associated with poorer academic outcomes (Duckworth and Seligman, 2005), substance abuse (De Wit, 2009), risky sexual behavior (Charnigo *et al.*, 2013) and a myriad of other negative life outcomes. What is more, the most severe manifestations of impulsive behavior, such as those present in disorders like psychopathy, substance abuse disorder and attention-deficit/hyperactivity disorder (ADHD), are estimated to cost nearly one trillion dollars annually in the USA alone from criminal social costs, medical care costs and losses in economic productivity (Kiehl and Hoffman, 2011). Given the negative impact that impulsivity has on both individual and societal well-being, it has therefore been of interest to elucidate the neurobiological underpinnings of impulsive behavior, with the hope that this knowledge can assist in identifying individuals who are at-risk for exhibiting impulsive behavior, as well as lead to better treatment options.

 Substantial progress has been made over the past few decades in identifying the neural substrates whose integrity is related to individual differences in impulsivity. Across numerous species, and in both clinical and healthy samples, studies have highlighted a distinct set of relevant brain regions, connections and neurotransmitter systems in reward-processing and decision-making circuitry whose integrity relates to levels of impulsivity (Buckholtz *et al.*, 2010; Dalley *et al.*, 2011; Dalley and Robbins, 2017). This circuitry involves gray matter nodes in the ventral midbrain, basal ganglia, thalamus, motor cortices and prefrontal cortex, the white matter connections between these nodes and the monoamine neurotransmitter systems (Buckholtz *et al.*, 2010; Dalley *et al.*, 2011; Dalley and Robbins, 2017).

Yet, despite the identification of a network of neural substrates whose integrity is associated with individual differences in impulsivity, there remains a lack of consensus regarding the direction of these relationships and the precise nature of aberrance associated with impulsive behavior (e.g. hyper- or hypo-connectivity? Excessive or diminished gray matter? etc.) (Colzato *et al.*, 2009; Lee *et al.*, 2009; Ghahremani *et al.*, 2012; Kayser *et al.*, 2012; Robertson *et al.*, 2015; Angelides *et al.*, 2017; Korponay *et al.*, 2017a). A further unresolved question is whether the nature of this aberrance is different by degree or by kind when comparing relatively high, but subclinical, impulsivity in the general population to clinical impulsivity in neuropsychiatric disorders. For instance, if high levels of impulsivity in the general population are associated with low levels of gray matter in the prefrontal cortex, would severe levels of impulsivity in a clinical population be associated with even lower levels of gray matter volume in the prefrontal cortex? Or are the neural correlates of severe impulsivity in clinical populations distinct from those in the general population?

 To address this question, here we conduct systematic reviews of the literature studies on the relationship between impulsivity and gray matter in the general population and in severely impulsive individuals with psychopathy. Psychopathy is a mental health disorder characterized by callous lack of empathy and impulsive-antisocial behavior. Present in roughly one-fourth of adult prison inmates, psychopathy is associated with a disproportionately high incidence of violent crime, substance abuse and recidivism (Smith and Newman, 1990; Hare, 2003). The detrimental behaviors committed by psychopathic individuals, estimated to result in $460 billion annual costs in the USA alone (Kiehl and Hoffman, 2011), have been linked to the impulsive-antisocial aspects of the disorder (Walters, 2003; Edens *et al.*, 2008).

### Comparing the measurement of impulsivity in the general population and in psychopathy

Different instruments have been used in the general population and psychopathy literature studies to measure impulsivity with respect to neuroimaging assessments of gray matter structure. This section will detail the most common instrument used in each literature—the Barratt Impulsiveness Scale (BIS-11) (Patton and Stanford, 1995) and the Factor 2 dimension of the Psychopathy Checklist-Revised (PCL-R) (Hare, 2003)—and establish a basis with which to compare them.

#### General population.

Several self-report instruments and a host of task-based measurements are used in the literature to measure impulsivity and its different dimensions in healthy samples. The instrument that has been most frequently used in conjunction with structural neuroimaging in healthy samples is the BIS-11, which, as such, is the instrument of focus in this review. The BIS-11 is a self-report questionnaire containing 30 questions, each of which requires the subject to choose between ‘Rarely/Never’, ‘Occasionally’, ‘Often’ and ‘Almost Always’. Items are scored from 1 to 4. The BIS-11 yields a total score and three subscale scores derived by factor analysis: attentional impulsivity (e.g. ‘I am restless at the theatre or lectures‘), motor impulsivity (e.g. ‘I do things without thinking’) and non-planning impulsivity (e.g. I am more interested in the present than the future;) (Patton *et al.*, 1995). Higher scores indicate higher levels of impulsivity.

#### Psychopathy.

While several instruments exist for the measurement of psychopathy, the most commonly used in forensic populations with pathological levels of impulsivity is the PCL-R. The PCL-R includes both a structured clinical interview and a file review, which raters use to score an individual as either a 0, 1 or 2 on 20 trait items. As such, total scores on the PCL-R can range from 0 to 40. Individuals scoring between 0 and 20 are considered to be non-psychopathic, those scoring between 21 and 29 are considered to be intermediate, and those scoring 30 and above are considered to have psychopathy (Hare, 2003). Importantly, the PCL-R’s 20 trait items can be separated into two factors that reflect the callous/unemotional (Factor 1) and impulsive/antisocial (Factor 2) dimensions of the disorder (Harpur *et al.*, 1989). Therefore, Factor 2 score on the PCL-R is the most commonly used measurement to gauge impulsivity in individuals with psychopathy.

 It is important to underscore that Factor 2 score on the PCL-R is not a ‘pure’ measure of impulsivity. The items that comprise the Factor 2 dimension of the PCL-R are determined by factor analysis of the PCL-R items (Harpur *et al.*, 1989), and index a broad array of antisocial traits and behaviors that include some more explicitly related to impulsivity (e.g. ‘impulsivity’, ‘poor behavioral control’ and ‘need for stimulation’) and some more complex behaviors and life events that may indirectly index impulsivity (e.g. ‘sexual promiscuity’, ‘juvenile delinquency’, ‘revocation of conditional release’, ‘criminal versatility’, ‘lack of realistic long-term goals’, ‘irresponsibility’ and ‘many short-term marital relationships’). Furthermore, unlike the BIS-11, the PCL-R is not a self-report instrument.

#### Comparing BIS-11 scores with PCL-R Factor 2 scores.

Despite their differences, a study measuring both BIS-11 and PCL-R Factor 2 scores within the same sample demonstrates a significant positive correlation between the two (Snowden and Gray, 2011). This study of an incarcerated sample found that Factor 2 score was significantly positively correlated with BIS-11 total score (*R *= 0.26), BIS-11 motor impulsivity subscale score (*R *= 0.28) and BIS-11 non-planning impulsivity subscale score (*R *= 0.31), but not with BIS-11 attentional impulsivity subscale score (*R *= 0.05).

**Fig. 1.**
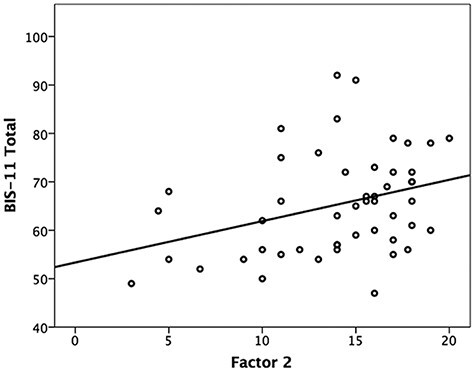
The within-subject relationship between PCL-R Factor 2 score and BIS-11 total score.

 To establish further basis to be able to compare BIS-11 and PCL-R Factor 2 scores, we corroborated the correspondence between these measures by administering the PCL-R and BIS-11 to a subset of an incarcerated sample (*n *= 49) used in our group’s recent prior studies of psychopathy (Korponay *et al.*, 2017b,c). Correlation analyses confirmed a significant positive relationship between a subject’s Factor 2 score and his/her BIS-11 total score of modest effect size (Table 1; Figure 1). In line with the prior study cited above, Factor 2 score was significantly correlated with BIS-11 total score, motor impulsivity subscale score and non-planning impulsivity subscale score, but it did not significantly correlate with attentional impulsivity subscale score.

**Table 1.** Relationship between PCL-R Factor 2 score and BIS-11 scores

	BIS-11 Total	BIS-11 Motor	BIS-11 Non-Planning	BIS-11 Attentional
Factor 2	*R* = 0.333 (*P* = 0.019)	*R* = 0.296 (*P* = 0.039)	*R* = 0.342 (*P* = 0.016)	*R* = 0.147 (*P* = 0.315)

Overall, prior work and the current data provide support to treat the two instruments as related measures of the construct of trait impulsivity, with PCL-R Factor 2 gauging more severe manifestations of impulsiveness than the BIS-11.

## Methods

### Systematic reviews

**Fig. 2.**
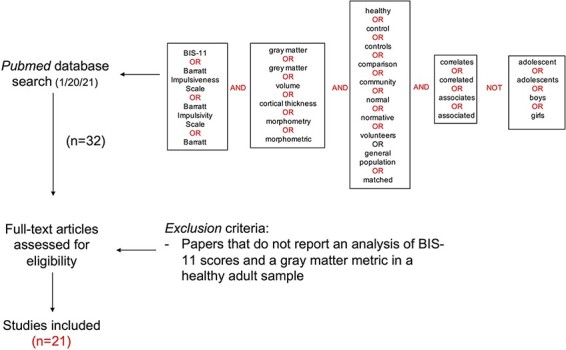
Systematic review search pipeline for identifying studies that examine the relationship between BIS-11 scores and gray matter in adults in the general population.

A systematic review of literature on the relationship between impulsivity and gray matter was first conducted for samples of the healthy, general population. The literature search aimed to identify all publications that met the following criteria: (i) study must report on the relationship between BIS-11 scores and a gray matter metric, and (ii) this relationship must be reported about a sample of healthy adults from the general population. Figure 2 shows the logical operators that were entered into PubMed to achieve initial filtering for these constraints.

The initial search yielded 32 qualifying peer-reviewed publications. From these 32, 11 publications were excluded after assessment of the full text, because they did not report an analysis of BIS-11 scores in relation to a gray matter metric in a healthy adult sample. This left a total of 21 publications for subsequent examination of findings in the general population literature.

**Fig. 3.**
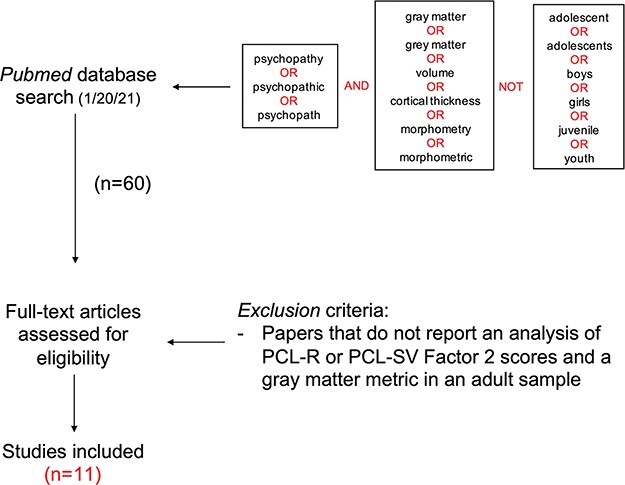
Systematic review search pipeline for identifying studies that examine the relationship between PCL-R Factor 2 scores and gray matter in adults with psychopathy.

 A systematic review was then conducted on the literature examining the relationship between impulsivity and gray matter in psychopathy. The literature search aimed to identify all publications that met the following criteria: (i) study must report on the relationship between Factor 2 scores on either the PCL-R or Psychopathy Checklist Screening Version (PCL-SV) and a gray matter metric, and (ii) this relationship must be reported in a sample of adults. Figure 3 shows the logical operators that were entered into PubMed to achieve initial filtering for these constraints.

The initial search yielded 60 qualifying peer-reviewed publications. From these 60, 49 publications were excluded after assessment of the full text, because they did not report an analysis of either PCL-R or PCL-SV Factor 2 scores in relation to a gray matter metric in a sample of adults. This left a total of 11 publications for subsequent examination of findings in the psychopathy literature.

## Results

### The relationship between gray matter volume and impulsivity in the general population

Twenty-one studies were found in the literature that examined the relationship between gray matter and impulsivity as measured by BIS-11 scores in samples of healthy adults from the general population. This literature converges on one modestly consistent pattern of findings that self-reported impulsivity in the general population as measured by the BIS-11 is associated with decreases in gray matter in the prefrontal cortex. This relationship is reported by eight studies (Matsuo *et al.*, 2009; Schilling *et al.*, 2012; Holmes *et al.*, 2016; Grodin *et al.*, 2017; Liu and Feng, 2017; Tu *et al.*, 2017; Korponay *et al.*, 2017a; Ai *et al.*, 2019), two of which also replicated the finding in independent samples (Holmes *et al.*, 2016; Liu and Feng, 2017). Eleven studies did not report a relationship between prefrontal gray matter and BIS-11 scores (Bjork *et al.*, 2009; Sala *et al.*, 2011; Deserno *et al.*, 2015; Mei *et al.*, 2015; Ide *et al.*, 2017; Qiu *et al.*, 2017; Kubera *et al.*, 2018; Besteher *et al.*, 2019; Li *et al.*, 2019; Rosenthal *et al.*, 2019; Zsido *et al.*, 2019), and two studies reported a positive relationship (Lee *et al.*, 2011; Cho *et al.*, 2013) (Table 2). The average sample size of the 10 samples reporting a negative impulsivity–gray matter relationship was 173, whereas the average sample size of the two samples reporting a positive relationship was 26. Within prefrontal cortex, the most consistent findings across studies were decreased gray matter in rostral and ventral prefrontal regions, including the orbitofrontal cortex. No relationships were observed between impulsivity and striatal gray matter.

**Table 2.** BIS-11 and gray matter associations in general population (healthy adults)

Study	*n*	Age	Impulsivity measure	Gray matter measure	Analysis	Relationship	Region
(Matsuo *et al.*, 2009)	62 (38 F)	35.4 ± 12.1	BIS-11	Volume	Regression	Negative	R, L OFC (total; motor and non-planning subscales)
(Bjork *et al.*, 2009)	29 (11 F)	37.4 ± 11.0	BIS-11	Volume	Regression	None	n/a
(Sala *et al.*, 2011)	15 (11 F)	34.2 ± 8.1	BIS-11	Volume	Regression	None (only hippocampus and dlPFC assessed)	n/a
(Lee *et al.*, 2011)	18 (0 F)	40.5 ± 7.5	BIS-11	Volume	Regression	Negative	L STG (attentional; motor)
						Positive	L OFC, L Lateral Frontopolar Cortex (non*-*planning)
(Schilling *et al.*, 2012)	32 (18 F)	35.2 ± 10.5	BIS-11 (German version)	Cortical thickness	Regression	Negative	L MFG (total, attentional, motor and non-planning subscales) OFC; R MFG (total and motor subscale)
(Cho *et al.*, 2013)	34 (11 F)	23.4 ± 4.3	BIS-11	Volume	Regression	Positive	L ACC; L DLPFC; R OFC; R, L medial PFC; R, L middle Cingulate (total; non-planning and attentional subscales)
(Deserno *et al.*, 2015)	24 (12 F) 26 (13 F)	27.29 ± 3.7 27.58 ± 3.7	BIS-11	Density	Group (high-impulsive *vs* low-impulsive)	None	n/a
(Mei *et al.*, 2015)	41 (17 F)	29.7 ± 10.1	BIS-11	Volume	Regression	None (only amygdala and hippocampus examined)	n/a
(Holmes *et al.*, 2016)	1015 (537 F)	21.4 ± 3.1	BIS-11	Cortical Thickness	Regression	Negative	R, L MFG; R, L ACC (motor subscale)
	219 (133 F)	21.2 ± 3.3	BIS-11	Cortical Thickness	Regression	Negative	R, L MFG; R, L ACC (motor subscale)
(Ide *et al.*, 2017)	113 (66 F)	32 ± 14	BIS-11	Volume	Regression	Positive	R, L parieto-occipital sulcus (total; attentional and non-planning subscales)
(Qiu *et al.*, 2017)	18 (0 F)	24.0 ± 3.08	BIS-11	Cortical thickness and subcortical volume	Regression	None	n/a
(Korponay *et al.*, 2017c)	105 (65 F)	48.6 ± 10.9	BIS-11	Volume	Regression	Negative	R Mofc (total; motor and non-planning) R, L paracingulate gyrus (non-planning subscale)
(Liu and Feng, 2017)	85 (55 F)	20.5 ± 2.07	BIS-11	Volume	Regression	Negative	L dlPFC, L calcarine gyrus (total)
						Positive	R STG (total)
	84 (51 F)	19.5 ± 1.4	BIS-11	Volume	Regression	Negative	L dlPFC, L MFG (total)
(Tu *et al.*, 2017)	56 (34 F)	33.9 ± 7.4	BIS-11 (Chinese version)	Cortical Thickness	Regression	Negative	L Inferior, middle and medial PFC (total)
(Grodin *et al.*, 2017)	49 (26 F)	38.8 ± 10.9	BIS-11	Volume Cortical thickness	Regression Regression	Negative Negative	L ACC (total) R, L ACC (total)
(Kubera *et al.*, 2018)	54 (38 F)	24.9 ± 4.02	BIS-11	Cortical thickness	Regression	Negative	L lingual gyrus; L superior temporal gyrus; R cuneus; R superior parietal gyrus (total) R, L pericalcarine gyrus (non-planning subscale)
(Ai *et al.*, 2019)	84 (43 F)	21.3 ± 1.7	BIS-11 (Chinese version)	Volume	Regression	Negative	R supplementary motor area; R paracentral lobule (total)
(Besteher *et al.*, 2019)	85 (57 F)	24.1 ± 3.0	BIS-11 (German version)	Volume	Regression	Positive	R inferior parietal gyrus; R postcentral gyrus; R supramarginal gyrus (total)
(Li *et al.*, 2019)	40 (2 F)	24.4 ± 3.6	BIS-11	Sulcal depth, gyrificationand cortical thickness	Regression	None	n/a
(Rosenthal *et al.*, 2019)	62 (23 F)	42.7 ± 11.69	BIS-11	Volume	Regression	None	n/a
(Zsido *et al.*, 2019)	29 (15 F)	21.9 ± 2.1	BIS-11	Cortical thickness	Regression	Positive	R, L middle temporal cortex; L transverse temporal cortex; (total)

### The relationship between gray matter and impulsivity in psychopathy

Eleven studies (two using the same sample) were found in the literature that examined the relationship between gray matter and impulsivity as measured by PCL-R or PCL-SV Factor 2 score in samples of adults. This literature converges on two consistent patterns of findings. The first is that impulsive-antisocial psychopathic traits are associated with increased gray matter in the striatum—five studies (Glenn *et al.*, 2010; Schiffer *et al.*, 2011; Cope *et al.*, 2012; Leutgeb *et al.*, 2015; Korponay *et al.*, 2017b) report this relationship (one of which found a local decrease in conjunction with a local increase (Cope *et al.*, 2012). This is consistent with the findings of a recent quantitative meta-analysis of gray matter in psychopathy (Deming and Koenigs, 2020). Within the striatum, the most consistent findings across studies are increased gray matter in the rostral caudate and the ventral striatum. The second pattern of findings is that impulsive-antisocial psychopathic traits are associated with widespread increases in gray matter throughout the prefrontal cortex—four studies (Cope *et al.*, 2012; Leutgeb *et al.*, 2015; Contreras-Rodriguez *et al.*, 2015b; Korponay *et al.*, 2017c) report this relationship (Table 3). Within prefrontal cortex, the most consistent findings across studies were increased gray matter in rostral and ventral regions, including the orbitofrontal cortex. The aforementioned quantitative meta-analysis did not reveal a consistent frontal cortical area where gray matter correlated with Factor 2 score (Deming and Koenigs, 2020). This is likely because the peak coordinates identified in the studies analyzed are spread throughout different areas of the frontal cortex, rather than concentrated in the same areas.

**Table 3.** PCL-R Factor 2 and gray matter associations in samples that include individuals with psychopathy

Study	Population	*N*	Age	Psychopathy Severity	Impulsivity Measure	Gray Matter Measure	Analysis	Relationship	Region
(De Oliveira-souza *et al.*, 2008)	Antisocial personality disorder (APD) patients	15 (7 F)	32 ± 14	Mean PCL-SV Score: 17.8 ± 3.8	PCL-SV Factor 2	Concent-ration	Regression (within the APD group)	None	n/a
	Healthy non-patients	15 (7 F)	32 ± 13	Mean PCL-SV Score: 0.4 ± 1.0					
(Muller *et al.*, 2008)	Psychopathy inpatients	17 (0 F)	33.0 ± 5.8	Mean PCL-R Score: 33.4 ± 4.1	PCL-R Factor 2	Volume	Regression	None (in temporal lobe)	Analysis for frontal cortical or striatal regions not reported
	Healthy non-patients	17 (0 F)	30.6 ± 5.9	Mean PCL-R Score: 0.5 ± 1.1					
(Glenn *et al.*, 2010)	Temporary employment agency sample	44 (4 F)	31.1 ± 6.7	Mean PCL-R Score: 20.1 ± 8.2	PCL-R Factor 2	Volume	Regression	Positive	Total striatum; L, R lenticular nucleus
(Schiffer *et al.*, 2011)	Non-offenders without SUDs	14 (0 F)	36.7 ± 11.4	Mean PCL-SV Score: 4.4 ± 2.6	PCL-SV Factor 2	Volume	Regression (across all subjects)	Quadratic: Negative for low scorers, Positive for high scorers	L nucleus accumbens; L insula
	Non-offenders with SUDs	13 (0 F)	37.3 ± 7.9	Mean PCL-SV Score: 6.5 ± 2.6					
	Violent Offenders without SUDs	12 (0 F)	37.4 ± 10.6	Mean PCL-SV Score: 9.3 ± 2.9					
	Violent Offenders with SUDs	12 (0 F)	36.4 ± 5.5	Mean PCL-SV Score: 12.8 ± 2.8					
(Cope *et al.*, 2012)	Community substance abuse sample	66 (30 F)	36.9 ± 7.9	Mean PCL-R Score: 18.4 ± 8.0	PCL-R Factor 2	Volume	Regression	Positive	Medial, inferior, middle, superior frontal gyri; ACC; postcentral gyrus; caudate head
								Negative	Temporal lobe; parahippocampal, fusiform gyri; insula; inferior parietal lobule; medial frontal gyrus; caudate tail
(Pujara *et al.*, 2014)	Incarcerated non-psychopaths	23 (0 F)	32.4 ± 8.0	Mean PCL-R Score: 14.1 ± 3.5	PCL-R Total	Volume	Regression (separate for each group)	None for non-psychopaths,	R nucleus accumbens
	Incarcerated psychopaths	18 (0 F)	32.2 ± 6.5	Mean PCL-R Score: 31.7 ± 1.7				Positive for psychopaths	R nucleus accumbens
(Ermer et al. 2013)	Incarcerated adult males	296 (0 F)	33.9 ± 9.5	Mean PCL-R Score: 21.3 ± 7.0	PCL-R Factor 2	Volume; Concentration	Regression	Negative	R Temporal Pole
(Contreras-Rodriguez *et al.*, 2015a)	Males with history of severe criminal offense	22 (0 F)	39.8 ± 9.2	Mean PCL-R Score: 27.8 ± 4.5	PCL-R Factor 2	Volume	Regression	Positive	Medial-dorsal PFC; medial PFC; R lateral PFC; operculum; cerebellum
(Leutgeb *et al.*, 2015)	Incarcerated adult males	40 (0 F)	38.1 ± 12	Mean PCL-R Score: 20.6 ± 7.7	PCL-R Factor 2	Volume	Regression	Positive	L, R OFC; L, R insula; R SMA; R putamen; L pallidum
(Korponay *et al.*, 2017c) and (Korponay *et al.*, 2017a)	Incarcerated adult males	124 (0 F)	31.6 ± 7.3	Mean PCL-R Score: 24.8 ± 7.1	PCL-R Factor 2	Volume	Regression	Positive	R mOFC; L middle and superior frontal gyri; L, R ventral striatum

A third observation is also suggested by this literature. Schiffer and colleagues (Schiffer *et al.*, 2011) report a quadratic relationship such that Factor 2 score is positively related to nucleus accumbens and insula volume in high PCL-SV scoring individuals but is negatively related to volume in these regions in low PCL-SV scoring individuals. Similarly, Pujara and colleagues (Pujara *et al.*, 2014) report that PCL-R total score is significantly positively related to nucleus accumbens volume within individuals with psychopathy, but not within individuals without psychopathy. These results are intriguing because they reflect the same overall difference in findings between the general population and psychopathy literature studies. Namely, they support the idea that the neural underpinnings of impulsivity in individuals with clinically significant levels of psychopathy and impulsive-antisocial traits may be distinct from those with high, but subclinical, levels of impulsive traits.

**Fig. 4.**
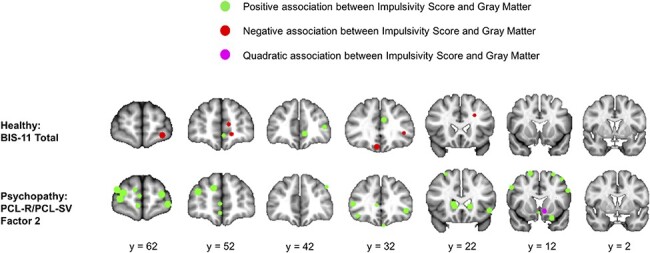
Peak coordinates reported by all included studies that conducted whole-brain voxel-wise analyses of the relationship between gray matter and either BIS-11 total score (for healthy subjects) or PCL-R/PCL-SV factor 2 score (for subjects with psychopathy).

Figure 4 shows the peak coordinates reported by all included studies that conducted whole-brain voxel-wise analyses of the relationship between gray matter and either BIS-11 total score (for healthy subjects) or PCL-R/PCL-SV Factor 2 score (for subjects with psychopathy). While these peak coordinates do not fully illustrate the findings from these literature studies (some studies do not conduct voxel-wise whole-brain analyses and thus do not report peak coordinates), they nonetheless reflect the overall pattern of findings. Of 11 frontal cortex peak coordinates reported in the healthy adult literature, the majority (seven) reflected a negative relationship between gray matter and BIS-11 total score. Of 22 frontal cortex peak coordinates reported in the psychopathy literature, all reflected a positive relationship between gray matter and PCL-R/PCL-SV Factor 2 score. Furthermore, while the healthy adult literature does not report any peak coordinates in the striatum, the psychopathy literature reports five (four positive and one quadratic).

#### Verifying effects specific to psychopathy within broader forensic samples.

An important caveat to these analyses is that, in nearly all studies in the psychopathy literature, the relationship between Factor 2 scores and gray matter volume is evaluated within samples that include lower PCL-R scoring, non-psychopathic individuals. As such, there is question as to whether the observed positive relationship between frontostriatal gray matter and impulsive-antisocial traits is truly specific to psychopathy. To address this, we cite several prior empirical observations, and present new analyses, that provide confidence that the positive gray matter-Factor 2 findings reported on in this review reflect, and are largely driven by, high PCL-R-scoring individuals with psychopathy.

First, the average PCL-R scores in the samples examined in this review are substantial: 33.4 ± 4.1, 20.1 ± 8.2, 18.4 ± 8.0, 31.7 ± 1.7, 21.3 ± 7.0, 27.8 ± 4.5, 20.6 ± 7.7 and 24.8 ± 7.1. This means that the examined samples are largely comprised of high PCL-R scoring individuals with scores close to or within the PCL-R scoring ranges used to classify psychopathy (PCL-R > 23; PCL-R > 26; PCL-R > 30) or intermediate/moderate psychopathy (30 > PCL-R > 20). As such, findings from analyses of these samples largely reflect relationships within moderate-to-high psychopathic individuals, despite the inclusion of a contingent of lower PCL-R scoring individuals. Furthermore, even forensic samples comprised of individuals with relatively low PCL-R scores (e.g. mean PCL-R = 14.5) (Gonsalves *et al.*, 2013) have significantly higher psychopathic traits than the general population (Blonigen *et al.*, 2003).

This notion is further supported, first, by the prior studies that allow separate dissection of effects for low PCL-R/SV scoring and high PCL-R/SV scoring individuals. As discussed, one such study found a quadratic relationship between PCL-SV Factor 2 score and nucleus accumbens volume, whereby the relationship was positive for high scorers and negative for low scorers. Similarly, in another study, PCL-R total score was significantly positively correlated with nucleus accumbens volume in high PCL-R scoring individuals with psychopathy but not in low PCL-R scoring individuals without psychopathy.

A re-analysis of data from Korponay and colleagues (Korponay *et al.*, 2017b,c) presented here provides stronger evidence that the positive frontostriatal GMV–Factor 2 relationship observed in forensic samples reflects, and is driven most strongly by, the high-scoring individuals with psychopathy. For each significant (*P* < 0.05) relationship reported in these studies between regional frontostriatal GMV and Factor 2 score, we re-analyzed the regression analysis separately within low PCL-R scoring non-psychopathic individuals (PCL-R < 20; *n* = 35) and within high PCL-R scoring individuals with psychopathy (PCL-R > 30; *n* = 41). Table 4 displays the results.

**Table 4.** Relationships between factor 2 score and gray matter volume in psychopathic and non-psychopathic individuals

Region	Group	Standardized Beta	Relationship	*P*-value
*Superior Frontal Gyrus (L)*	**Psychopathic**	**0.446**	**Positive**	0.004**
	Non-psychopathic	0.188	Positive	0.316
*Superior Frontal Gyrus (R)*	**Psychopathic**	**0.334**	**Positive**	0.013*
	Non-psychopathic	0.120	Positive	0.425
*Middle Frontal Gyrus (R)*	**Psychopathic**	**0.412**	**Positive**	0.002**
	Non-psychopathic	0.077	Positive	0.602
*Middle Frontal Gyrus (L)*	**Psychopathic**	**0.465**	**Positive**	0.005**
	Non-psychopathic	0.269	Positive	0.103
*Medial Orbitofrontal Cortex (R)*	Psychopathic	0.186	Positive	0.092
	Non-Psychopathic	0.136	Positive	0.350
*Putamen (R)*	**Psychopathic**	**0.342**	**Positive**	0.032*
	**Non-psychopathic**	**0.429**	**Positive**	0.030*
*Caudate (R)*	**Psychopathic**	**0.369**	**Positive**	0.040*
	Non-psychopathic	0.298	Positive	0.112
*Nucleus Accumbens (L)*	Psychopathic	0.225	Positive	0.192
	Non-psychopathic	0.172	Positive	0.405
*Nucleus Accumbens (R)*	Psychopathic	0.228	Positive	0.168
	Non-psychopathic	0.253	Positive	0.229

As in the original analyses, these analyses control for age, race, substance use, brain volume and PCL-R factor 1 scores. Bolding indicates significance at the

** *P* < 0.01 or

* *P* < 0.05 level.

Nine frontostriatal regions displayed a significant relationship between regional gray matter volume and Factor 2 score across the whole forensic sample. Among these, the positive GMV–Factor 2 relationship was significant in the psychopathy group for six regions. In contrast, the GMV–Factor 2 relationship was only significant in the non-psychopathy group for one region. These findings demonstrate that the positive frontostriatal GMV–Factor 2 relationships observed in the larger forensic sample are primarily driven by and reflect the relationships as they exist in high PCL-R scoring individuals with psychopathy. Together with the two prior studies that demonstrate a similar effect in forensic samples, and the high average PCL-R scores across all forensic samples in this literature, there is appreciable evidence to suggest that the positive GMV–Factor 2 relationship observed in forensic samples throughout the literature reflects the relationship as it exists in psychopathy.

## Discussion

Is severe impulsivity in psychopathy associated with a neural profile that is an extreme but continuous manifestation of the neural profile associated with high impulsivity in the general population (i.e. different by degree)? Or is it associated with a neural profile that is discontinuous and unique (i.e. different by kind)? Here, we conducted two systematic reviews—examining the relationship between impulsivity and gray matter in psychopathy and in the general population, respectively—and compared their findings. The resulting pattern of findings from these literature studies suggests that the neural profile associated with extreme impulsivity in psychopathy (increased gray matter in rostral and ventral areas of the striatum and prefrontal cortexes) is different by kind from that associated with high impulsivity in the general population (decreased gray matter in the rostral and ventral prefrontal cortexes). These results suggest that severe impulsivity in psychopathy may arise from a pathophysiological mechanism that is unique to the disorder and which is separable from the mechanisms resulting in relatively high, but subclinical, impulsivity in the general population. The results caution against the use of community samples to examine impulsive psychopathic traits in relation to neural correlates, where the relationship between impulsivity and underlying neurobiology may be an inaccurate model of the relationship in clinical-level cases of psychopathy. These findings also prompt the need for future studies designed to directly test the effect of group on the relationship between impulsivity and gray matter volume in samples comprised of healthy individuals and individuals with psychopathy.

An important question for future research is to determine the neurobiological mechanisms by which greater gray matter volume may underlie impulsivity in psychopathic individuals, whereas reduced gray matter may underlie impulsivity in the general population. Intra-individual changes in gray matter levels have been attributed to a range of prenatal, perinatal and postnatal developmental processes including neuron development, synaptic pruning, myelination, dendritic length changes and changes in capillary and glial cell levels (Gogtay *et al.*, 2004; Courchesne *et al.*, 2007; Giorgio *et al.*, 2010; Anderson, 2011; Gilmore *et al.*, 2012). Aberrance in one or more developmental processes could therefore lead to gray matter levels that are either greater (e.g. via deficient synaptic pruning) or lesser (e.g. via insufficient neuron development) than what is optimal for efficient neural processing, leading to cognitive deficit in either case. Our findings suggest that impulsivity may arise from distinct neurodevelopmental cascades in psychopathy and in the general population.

At present, the literature on the relationships between impulsivity and other neurobiological metrics (e.g. functional connectivity, dopaminergic functioning, etc.) in healthy adults and psychopathy is too small to permit a meaningful comparison of findings. As studies on these metrics grow, it will be of interest to examine whether there is a similar ‘difference by kind’ distinction in these metrics as well. Nonetheless, at least one example of disorder-specific, ‘different by kind’ functional neural correlates of impulsivity can be found in the literature on ADHD. A meta-analysis of fMRI data found that while the ventral striatum shows decreased activity during reward anticipation in individuals with ADHD, ventral striatal activity during reward processing is increased in subjects in the healthy population with high levels of trait impulsivity (Plichta and Scheres, 2014). However, as the psychopathy literature itself demonstrates, there is not necessarily correspondence between the neural correlates of a disorder as a whole and the neural correlates of a particular subcategory of symptoms of the disorder. Nearly all group analyses in the psychopathy literature that compare gray matter in a psychopathy group to a non-psychopathy group find decreased prefrontal gray matter in the psychopathy group (Yang *et al.*, 2005; De Oliveira-souza *et al.*, 2008; Muller *et al.*, 2008; Ly *et al.*, 2012; Contreras-Rodriguez *et al.*, 2015a), despite the findings highlighted here of increased prefrontal gray matter with increasing impulsive-antisocial traits in psychopathy. Indeed, one study reported both of these findings within the same sample (Contreras-Rodriguez *et al.*, 2015a). The discrepancy may be attributable to the callous/unemotional (Factor 1) dimension of psychopathy being typically associated with gray matter decreases (De Oliveira-souza *et al.*, 2008; Contreras-Rodriguez *et al.*, 2015a). Overall, this demonstrates the need for greater future examination of the neural correlates specifically of impulsivity in individuals with disorders such as ADHD, before being able to reliably compare findings to those in the present report.

## Limitations

As previously discussed, the instruments used to measure impulsivity in the general population and in psychopathy are different. The BIS-11 is a self-report instrument, while the PCL-R involves a structured interview and file review. Factor 2 of the PCL-R is also not a pure measurement of impulsivity. Future studies on neural correlates of BIS-11 scores within psychopathy samples could help standardize comparisons to findings in the general population. For now, prior reports (Morgan *et al.*, 2011; Snowden and Gray, 2011) and the new data presented here on the significant positive correlation between BIS-11 and PCL-R Factor 2 score provide a provisional basis to compare findings across the two literature studies (See also **Supplementary Data**).

 A second limitation is that both of these literature studies are still somewhat small. Furthermore, only a subset of studies in these small literature studies reports peak coordinates from voxel-wise analysis, precluding the ability to perform a quantitative meta-analysis. And despite the convergence of findings in both literature studies around several identifiable patterns, the findings are not unanimous amongst all studies in each literature. As such, more studies of neural correlates of impulsivity within both populations are needed to further verify whether this relationship is indeed distinct in each population.

 Third, whereas all the studies considered here from the general population literature include samples that are close to evenly split between males and females, the majority of the psychopathy literature contains predominantly male samples. While the authors are not aware of any reports detailing distinct neural correlates of impulsivity for males and females, this is an important consideration to keep in mind when comparing the two literature studies.

## Conclusion

Overall, this study suggests that there may be important neurobiological signatures that distinguish high, but subclinical, levels of impulsivity from extreme, clinical levels of impulsivity. In particular, subclinical impulsivity in the general population and clinical impulsivity in psychopathy appear to have underlying neural correlates that are different by kind, rather than by degree. The results caution against the use of community samples to examine impulsive psychopathic traits in relation to neural correlates, where the relationship between impulsivity and underlying neurobiology may be an inaccurate model of the relationship in clinical-level cases of psychopathy.

## Supplementary Material

nsab045_Supp
