# Supplementary material for: Gray matter correlates of impulsivity in psychopathy and in the general population differ by kind, not by degree: a comparison of systematic reviews
Source: Soc Cogn Affect Neurosci. 2021 Apr 9;16(7):683–95. doi: 10.1093/scan/nsab045 (PMC8259272; doi:10.1093/scan/nsab045)
Supplement: nsab045_Supp [file nsab045_supp.zip › SUPPLEMENTARY DATA.docx]

**SUPPLEMENTARY DATA**

**Comparing BIS-11 and PCL-R Factor 2 Relationships with Gray Matter in the Same Sample**

As we discuss, there are important differences between the BIS-11 and PCL-R Factor 2 measurement scales. As such, it is important to consider whether differences in these scales may contribute to the distinct relationship found between gray matter and impulsivity in psychopathic individuals and in the general population.

In the manuscript we cite prior literature (Snowden and Gray 2011), and provide new data from our sample, to demonstrate that there is a statistically significant positive correlation of moderate effect size between PCL-R Factor 2 score and BIS-11 total score. Given that both scales measure similar concepts, and given that, quantitatively, they are significantly positively linked, it seems unlikely that using one scale versus the other would produce the stark opposite-direction findings (i.e. positive vs. negative relationships) observed in the two literatures.

To test this supposition, we examined the relationship between BIS-11 total score and gray matter in the subset of our sample (Korponay et al. 2017a, 2017b) that had both PCL-R Factor 2 scores and BIS-11 total scores (n=11 non-psychopathic individuals; n=18 individuals with psychopathy). These regressions were run on the same nine brain regions found to display a significant positive relationship between regional gray matter volume (GMV) and PCL-R Factor 2 score. Here, Factor 2 score was replaced with BIS-11 total score.

**Supplemental Table 1.** Relationships Between BIS-11 Total Score and Gray Matter Volume

| **Region** | **Group** | **Standardized Beta** | **Relationship** | **p-value** |
| --- | --- | --- | --- | --- |
| *Superior Frontal Gyrus (L)* | Psychopathic | 0.406 | Positive | 0.298 |
|  | Non-Psychopathic | 0.191 | Positive | 0.589 |
|  | | | | |
| *Superior Frontal Gyrus (R)* | Psychopathic | 0.081 | Positive | 0.804 |
|  | Non-Psychopathic | 0.056 | Positive | 0.881 |
|  | | | | |
| *Middle Frontal Gyrus (R)* | Psychopathic | 0.423 | Positive | 0.189 |
|  | Non-Psychopathic | 0.114 | Positive | 0.756 |
|  | | | | |
| *Middle Frontal Gyrus (L)* | Psychopathic | 0.366 | Positive | 0.336 |
|  | Non-Psychopathic | 0.218 | Positive | 0.499 |
|  | | | | |
| *Medial Orbitofrontal Cortex (R)* | Psychopathic | 0.355 | Positive | 0.251 |
|  | Non-Psychopathic | 0.104 | Positive | 0.741 |
|  | | | | |
| *Putamen (R)* | Psychopathic | 0.338 | Positive | 0.460 |
|  | Non-Psychopathic | 0.548 | Positive | 0.275 |
|  | | | | |
| *Caudate (R)* | Psychopathic | 0.500 | Positive | 0.311 |
|  | Non-Psychopathic | -0.059 | Negative | 0.858 |
|  | | | | |
| *Nucleus Accumbens (L)* | Psychopathic | 0.184 | Positive | 0.681 |
|  | Non-Psychopathic | 0.000 | Positive | 0.999 |
|  | | | | |
| *Nucleus Accumbens (R)* | Psychopathic | 0.361 | Positive | 0.421 |
|  | Non-Psychopathic | 0.016 | Positive | 0.975 |

As in the original analyses, these analyses control for age, race, substance use, brain volume, and PCL-R factor 1 scores.

The results show that all relationships between GMV and BIS-11 total score in psychopathic individuals are positive, just like the relationships between GMV and PCL-R Factor 2 scores in these individuals. Moreover, the effect sizes are similar to the PCL-R Factor 2 data (although the findings for the BIS-11 data do not reach statistical significance, due to the considerable reduction in sample size). Furthermore, the weaker coupling between GMV and BIS-11 scores in the low PCL-R scoring non-psychopathic group relative to the psychopathic group mirrors the findings from the PCL-R Factor 2 regressions. Altogether, these findings, coupled with the present and prior reports of a significant positive correlation between PCL-R Factor 2 scores and BIS-11 total scores, suggest that differences between the PCL-R and BIS-11 contribute minimally to the opposite-direction findings observed between the general population and psychopathy literatures.

**References**

Korponay, C., et al. (2017a), 'Impulsive-antisocial dimension of psychopathy linked to enlargement and abnormal functional connectivity of the striatum', *Biol Psychiatry Cogn Neurosci Neuroimaging,* 2 (2), 149-57.

--- (2017b), 'Impulsive-antisocial psychopathic traits linked to increased volume and functional connectivity within prefrontal cortex', *Soc Cogn Affect Neurosci,* 12 (7), 1169-78.

Snowden, R. J. and Gray, N. S. (2011), 'Impulsivity and psychopathy: associations between the barrett impulsivity scale and the psychopathy checklist revised', *Psychiatry Res,* 187 (3), 414-7.
